# Management of headache and associated factors among undergraduate medicine and health science students of University of Gondar, North West Ethiopia

**DOI:** 10.1186/s10194-016-0647-4

**Published:** 2016-05-23

**Authors:** Eshetie Melese Birru, Zenahebezu Abay, Mohammedbrhan Abdelwuhab, Abebe Basazn, Betelhem Sirak, Fitsum Sebsibe Teni

**Affiliations:** Department of Pharmacology, College of Medicine and Health Sciences, University of Gondar, PO Box: 196, Chechela Street, Lideta subcity Kebele 16, Gondar, Ethiopia; Department of internal Medicine, College of Medicine and Health Sciences, University of Gondar, Chechela street, Lideta subcity kebele 16, Gondar, Ethiopia; Unit of Pharmacognosy, College of Medicine and Health Sciences, University of Gondar, Chechela street, Lideta subcity kebele 16, Gondar, Ethiopia; Department of Pharmaceutics and Social Pharmacy, College of Health Sciences, Addis Abeba University, Lideta Subcity, Churchil Avenue, Addis Ababa, Ethiopia

**Keywords:** Headache, Migraine, Analgesics, Pharmacoepidemiology, Student

## Abstract

**Background:**

The headache disorders, namely, migraine and tension type headache and the associated analgesic consumption is badly underestimated and thus makes a major current public health problem. The objective of this study was to determine the prevalence of migraine and tension type headaches and the associated management options used among undergraduate students of College of Medicine and Health Sciences, University of Gondar, Gondar, Ethiopia.

**Method:**

Institution based cross sectional study was conducted among 720 students in May, 2014. Pretested and structured self-administered questionnaires were used as data collecting tool followed by short interview to diagnose the type of headache based on the International Headache Society diagnostic criteria. SPSS version 20 was also used to analyse the data descriptively as well as inferentially using logistic regression models to investigate factors associated with presence of headache and analgesic use.

**Result:**

The prevalence of lifetime headache and headache in the last 12 months was 81.11 and 67.22 %, respectively. Migraine and tension type headache were having 94 (13.06 %) and 481 (66.81 %) prevalence, respectively. Prevalence of life time headache was significant among females, students with family history of headache and lack of adequate vacation time. Similarly, lifetime prevalence of analgesic use for headache was 72.45 % and it had statistical association with sex, age, type of headache, lack of adequate vacation time and family history of headache. Majority of the students, migraineurs (54.65 %) and the tension type headache sufferers (66.17 %) commonly used paracetamol.

**Conclusion:**

High prevalence without adequate medical care seeking behaviour and the associated significant analgesic consumption necessitate the designing of all rounded strategies to improve the quality of life of individuals with such neurologic disorders.

## Background

Headache disorders, mainly the primary including migraine, tension type and cluster, are considered as major global health problems due to their high prevalence, chronicness and their substantial disability burden upon the sufferers [1, 2]. The socioeconomic impact of headache is worsened by sufferers’ low tendency of seeking medical care for their headache despite the high prevalence [3, 4]. The global burden of headache is very large [5, 6] but paradoxically, a burden of headache widely ignored [7] which will still increasingly affects quality of life and routine activities [8, 9]. According to the Global Burden of Disease Survey 2010, headache disorders are among the top 10 causes of disability worldwide [2, 10]. Economic burden of headache is substantial especially due to its peak prevalence in the most productive years of life [3] and results from work-related disability than the direct medical cost for treating it [11, 12].

The International Classification of Headache Disorders (ICHD-IIIβ) divides headaches into primary and secondary forms [13]. These painful conditions are related to a major lack of productivity at work, limitation of social activities and impairment of quality of life [14–16].

Recurrent headache is a risk factor for future chronic headache and other pain syndromes [17]. The risk of developing headache is greater in individuals with a family history, smoking, high body mass index, sleeping problems, substance abuse, oversleeping, premenstrual period, stressful life events, hot/cold weather, menstruation, hanger and others [1, 18–20].

Headache disorders especially in student populations are usually under-diagnosed and under-treated conditions and thus the headache attacks lead to lose of days of study and worse academic performance [21]. Headache is often treated with analgesics and is the most common reason for analgesic use in the general population [22]. In the current scenario of the increased prevalence of headache, most of the victims have been found to practice self-medication leading to irrational treatment and even possibly induction of refractory type of headache as well as analgesic over use headache [23]. Very high percentage (88.2 %) of students was reported to take over the counter drugs without the consultation of physicians in most countries [24]. Thus, the concern of drug safety will increase with regard to the possible contraindications, manner of dosing and drug interactions of analgesics [25].

Paracetamol, non-steroidal anti-inflammatory drugs (NSAIDs) and opioids are among the drugs that are most often implicated in serious or fatal medication errors [26]. Thus, improved understanding of the types of headache and its course of illness, especially migraine due to its complexity has led to the provision of appropriate health care services which can bring immediate relief of episodes of headache.

There is a scanty data on the prevalence of primary headaches in sub-Saharan Africa in general and Ethiopia in particular, even if it is stated that the prevalence of headaches is low as compared to Europe and North America [5]. Headache is a highly prevalent condition among students at University than other population [18] but the associated analgesic consumption has not been well estimated. Taking all the aforementioned issues of headache and the absence of previous reports on headache in student populations of Ethiopia, this study is conducted to assess the prevalence, precipitating factors, and management options of headache among undergraduate medicine, pharmacy, and other health science students of University of Gondar, Ethiopia. In addition to financial restriction and man power shortage, this section of students are selected for this study since we supposed that there is high educational burden in these students than others that may predispose them for frequent headache attacks.

## Methods

### Study design and period

Institution based cross sectional study was used to determine the prevalence of headache, to evaluate management options of headache and associated factors including the impact of headache and its precipitating factors among College of Medicine and Health Sciences, University of Gondar regular undergraduate students in May, 2014. University of Gondar is the oldest university in Ethiopia and served the country for more than half a century. Home of the university, Gondar City has an average temperature of 18–22 °C and located 738 km away from Addis Ababa, the capital city of Ethiopia, to the Northwest direction. At the time of study, 2013/2014 academic year about 3270 regular undergraduate students coming from each corner of the country were enrolled in College of Medicine and Health Science.

### Sample size determination

Using Epi Info Statcalc program the assumptions made for the sample size calculation were: a single population proportion formula with a 95 % confidence interval, a proportion of 50 % and worst acceptable value 53.5 % (i.e. 3.5 % margin of error). Twenty percent (20 %) was added for non-response and other contingencies and finally the sample size became 759.

### Sampling procedure

In this study, considering stream of study and year of enrolment multistage stratified sampling was used.. After obtaining the verbal informed consent each of the students were randomly selected when they were attending class. Since as usual they were simply on clinical practice without active teaching learning process year VI medical students were excluded in this study.

### Data collection

The data collecting tool was checked and evaluated for its appropriateness by doing pre-test on 30 students of College of Natural and Computational Science, a separate campus of University of Gondar. After half a day training of nurses by neurologist on the diagnostic criteria of International Headache Society (IHS) data was collected from the randomly selected students under the supervision of the investigators using English version structured self administered questionnaire. Students with a positive history of headache were interviewed further to diagnose the type of headache they experienced as per the IHS diagnostic criteria [10] and to exclude headache other than primary headache. The first part contains socio-demographic characteristics of students. The second part of the questionnaire was about the presence of headache, followed by questions specific for headache sufferers like number of headache episodes per time period and impact of headache on daily activity of students. The third and fourth parts of the questionnaire were about triggering factors of headache and experience of subjects on the management of headache, respectively.

### Data processing and analysis

After proper cleaning, cross checking and coding of data descriptive as well as inferential statistics of the study was analysed by using SPSS version 20. Bivariate and multivariate logistic regression was used to identify factors associated with prevalence of headache and drug use for headache among students. The variables with *p* value less than 0.5 in the bivariate analysis were entered to the multivariate model using the enter regression method. Model fitness was checked using Hosmer and Lemeshow goodness of a fit test (*P* = 0.63 and *p* = 0.68, respectively). Operationally, respondents who had taking medications more than three times of the full dose of each of the medications in a week were considered as frequent drug users.

### Data quality control

Beyond Pretesting the questionnaire and training of the data collectors, the data collection process was closely monitored and supervised by investigators. Since there was interview for students with positive history of headache it made the exclusion of headache other than primary headache more efficient. The collected data were reviewed and checked for completeness before data entry and incomplete data were discarded.

### Ethical issues

Ethical clearance was requested and obtained from the Institute Review Board (IRB) of the University of Gondar. In addition, before the structured questionnaires were distributed to the students at the beginning of class the verbal informed consent was obtained from the teacher in charge and respondents who were selected to participate in the study.

## Result

From the total of 759 drawn students, only 720 of them completed their interview and self administered questionnaire properly, giving response rate of 94.86 %. At the time of the study, the majority of the students of College of Medicine and Health Sciences, University of Gondar were males, 2364 (72.29 %) which was having the same composition of participants involved in this study. This consistent composition was also maintained for year of enrolment and stream of study of our study population.

### Sociodemographic characteristics of the study population

Most respondents of this study were males, 520 (72.22 %). And the average age of the participants was 21 (±1.61) years. Furthermore, as it is shown in Table 1 majority of the participants were Amhara in their ethnicity and Orthodox Christian in their religion, 382 (53.06 %) and 499 (69.31 %), respectively.

**Table 1:** Regressional analysis of factors associated with life time prevalence of Headache among students of College of Medicine and Health Sciences, University of Gondar, Ethiopia (*n* = 720), May, 2014

Variable		Life time headache		Total (*n*, %)	OR with 95 % CI	
		Yes	Yes		Crude	Adjusted
Sex	Male	408	112	520 (72.22 %)	1	1
	Female	176	24	200 (27.78 %)	2.013 (1.252–3.237)	**2.722 (1.575–4.702)**
Age	18–21	208	56	264 (36.67 %)	0.743 (0.459–1.201)	1.271 (0.534–1.725)
	22–25	216	48	264 (36.67 %)	0.900 (0.550–1.472)	1.423 (0.817–1.921)
	>25	160	32	192 (26.67 %)	1	1
Ethnicity	Amhara	300	82	382 (53.06 %)	1	1
	Oromo	87	17	104 (14.44 %)	1.399 (0.788–2.484)	1.635 (0.790–3.386)
	Others	197	37	234 (32.5 %)	1.455 (0.949–2.232)	1.449 (0.891–2.358)
Religion	orthodox	400	99	499 (69.31 %)	1	1
	Muslim	72	15	87 (12.08 %)	1.188 (0.653–2.161)	0.88 (0.408–1.601)
	Protestantism	80	14	94 (13.06 %)	1.414 (0.769–2.600)	0.774 (0.353–1.699)
	Others	32	8	40 (5.56 %)	0.990 (0.442–2.215)	0.621 (0.254–1.518)
Family history	Yes	251	27	278 (38.61 %)	3.043 (1.936–4.782)	**4.049 (2.465–6.653)**
	No	333	109	442 (61.39 %)	1	1
Department	Medicine	176	32	208 (28.89 %)	1.031 (0.672–1.583)	1.118 (0.653–1.914)
	Pharmacy	111	16	127 (17.64 %)	1.089 (0.657–1.806)	1.166 (0.638–2.131)
	Others	297	88	385 (53.47 %)	1	1
Year of study	I	150	30	180 (25.00 %)	1.698 (0.875–3.295)	2.132 (0.870–5.229)
	II	137	29	166 (23.06 %)	1.604 (0.823–3.130)	1.595 (0.684–3.717)
	III	128	34	162 (22.50 %)	1.279 (0.664–2.461)	1.090 (0.471–2.519)
	IV	116	25	141 (19.58 %)	1.576 (0.792–3.134)	1.123 (0.503–2.506)
	V	53	18	71 (9.86 %)	1	1
Lack of adequate vacation time	Yes	212	18	230 (31.94 %)	3.736 (2.213–6.308)	**3.863 (2.235–6.677)**
	No	372	118	490 (68.06 %)	1	1

Adjusted odds ratio in bold refers factors which do have statistical association with the dependent variable

### Prevalence of headache

The life time prevalence of primary headache among participants was 584 (81.11 %). From this, 94 (13.06 %) were having migraine, 481 (66.81 %) were having tension type headache and the remaining 9 (1.25 %) did not fulfil criteria for either migraine or tension-type headache according to the IHS diagnostic criteria. In addition, from the total of female students involved in this study 32 (16.00 %) were having migraine while among males it was only 62 (11.92 %) prevalent. Among migraineurs of participants 71 (75.53 %) were having family history of headache.

Headache in the last 12 months was 464 (67.22 %) prevalent in the study population. Among all these, the number of headache episodes was found <1per month, 1–2 per month, 2–3 per month and ≥1 per week in 96 (20.69 %), 232 (50.00 %), 72 (15.52 %) and 64 (13.79 %), respectively. During headache attacks among the participants 152 (32.76 %) never, 160 (34.48 %) rarely, 101 (21.77 %) usually and 51 (10.99 %) always need to limit or avoid their daily activity.

### Triggering factors of headache

As it is depicted in Table 2 stress/tension, too little sleep, reading for longer time, change in mood and some others are mentioned as they are the potential triggering factors of students’ headache.

**Table 2:** Triggering factors of headache among students of College of Medicine and Health Sciences, University of Gondar, Ethiopia (*n* = 584), May, 2014

Factor	Frequency	Percent
Intense light, smell and sound	264	45.21
Allergies	176	30.14
Stress/tension	472	80.82
Too little sleep	424	72.60
Too much sleep	85	14.04
Missed meals	120	20.55
Lack of caffeine	104	17.81
Too much caffeine	96	16.44
Entering a certain/strange place	128	21.92
Change in mood	320	54.78
Certain types of food	32	5.48
Watching TV for long hrs	224	38.36
Working on computers for long period	272	46.58
Menstrual period	96	16.44
Reading for longer time	352	60.27

### Management of headache

From the total of respondents who had headache ever 429 (72.45 %) of them used drugs for treatment of headache in their life time (Table 3). From the total of participants with migraine and tension type headache, 86 (91.49 %) and 337 (70.06 %) were having drug use experience for headache episodes, respectively. The association of some factors with life time drug use of migraine and tension type headache sufferers is shown in Table 4 below.

**Table 3:** Management options of headache used among students who had headache in the last 12 months, College of Medicine and Health Sciences, University of Gondar, Ethiopia (*n* = 464), May, 2014

Management options used	Frequency	Percent (%)
Take medications available from their own locker and/or share from friends	152	32.76 %
Purchase medications from pharmacy	208	44.83 %
Visit physician	32	6.90 %
Take substances other than drugs^a^	24	5.17 %
Nondrug treatments such as just lie down in quiet place, showering, Cold press	120	25.86 %

^a^substances like tea, coffee, leaves of ocimum sanctum and other traditional treatments

**Table 4:** Regressional analysis of factors associated with life time analgesic use for headache among students of College of Medicine and Health Sciences, University of Gondar, Ethiopia (*n* = 575), May, 2014

Variable		Life time Drug use		OR with 95 % C.I.	
		Yes	No	Crude	Adjusted
Sex	Male	271	136	1	1
	Female	152	16	4.768 (2.737–8.303)	**6.859 (3.678–12.791)**
Age	18–21	146	54	1.302 (0.826–2.052)	1.125 (0.678–1.866)
	22–25	169	46	1.769 (1.112–2.815)	**2.433 (1.429–4.141)**
	>25	108	52	1	1
Ethnicity	Amhara	216	78	0.929 (0.614–1.407)	1.531 (0.945–2.479)
	Oromo	61	25	0.813 (0.465–1.444)	1.108 (0.574–2.142)
	Others	146	49	1	1
Family history	Yes	203	48	1.999 (1.351–2.958)	**2.510 (1.592–3.958)**
	No	220	104	1	1
Department	Medicine	134	40	1.272 (0.823–1.965)	1.180 (0.719–1.936)
	Pharmacy	78	31	0.971 (0.598–1.576)	1.080 (0.598–1.950)
	Others	211+	81	1	1
Type of Headache	Migraine	86	8	4.593 (2.169–9.727)	**3.494 (1.589–7.686)**
	Tension Type	337	144	1	1
Lack of adequate vacation time	yes	170	37	2.088 (1.374–3.173)	**2.510 (1.592–3.958)**
	No	253	115	1	1

N. B. This statistical analysis was performed for subjects who were diagnosed as migraineur and tension type headache sufferer

Adjusted odds ratio in bold refers factors which do have statistical association with the dependent variable

Among students who had drug use experience for headache 275 (64.10 %) were using paracetamol, 106 (24.71 %) were using diclofenac, 32 (7.46 %) were using ibuprofen and the remaining 16 (3.73 %) were using migraine specific agents commonly. None of students were having the experience of using medications for the prophylaxis of headache Table 5

**Table 5:** Analgesics used/commonly used by migraineurs and tension type headache sufferers College of Medicine and Health Sciences, University of Gondar, Ethiopia (*n* = 464), May, 2014

Drugs	Migraine (*n* = 86)	Tension type (*n* = 337)
Paracetamol	47 (54.65 %)	223 (66.17 %)
Diclofenac	15 (17.44 %)	90 (26.71 %)
Ibuprofen	8 (9.30 %)	24 (7.12 %)
Migraine specific agents	16 (18.61 %)	–

.

Among drug users for headache, 72 (16.78 %) said that the medications they have taken commonly didn’t treat their headache effectively. In response to treatment failures, 8 (11.11 %) consulted health care professionals, 13 (18.06 %) took additional dose of the drug, 11 (15.28 %) changed their medication, and the remaining 40 (55.56 %) used non drug measures such as sleeping, relaxation, avoiding disturbance. In addition, from the total respondents having drug use experience 32 (7.46 %) had experience of using two or more analgesics simultaneously when their headache is being sever and/or less responsive for a single pain killer.

Similarly, from all drug users 192 (44.76 %) used drugs for headache frequently. Furthermore, from all those frequent drug users 144 (75.00 %) perceived that the drug brought different health problems from which 72 (50.00 %) was dependency, 32 (22.22 %) relapsing headache, 48 (33.33 %) adaptations, and the rest 8 (5.56 %) other untoward effects like GI disturbance and ulceration.

## Discussion

Even if mostly the health care providers as well as the concerned authorities didn’t give adequate attention due to its usually reversibility and mild to moderate severity, headache is becoming the most prevalent health problem and negatively affects quality of life. In the student population of this study the life time prevalence of headache was 81.11 % which is close to a community based report from Addis Ababa (83.1 %) [27] and Singapore (82.7 %) [28]. But the prevalence of headache in this study is higher than a report from undergraduate student headache prevalence of Southern Brazil 74.5 % [29] and lower than other reports from Brazil [30, 31] and Nigeria 88 % [32]. The last 1 year prevalence of headache in our study population was 67.22 % and this is still closer to the report from Addis Ababa (67.5 %) [27] and lower than the report from Brazil which was 91 % and the discrepancy might be due to methodological, study population or real difference [30].

Compared to the male counterparts the prevalence of headache among females students was found higher and accordingly the likelihood of headache among females compared to males was 2.72 times higher (AOR = 2.722; 95 % CI 1.252–3.237). Supporting this, a number of studies [20, 21, 33] demonstrated that prevalence of headache among females is higher than male counterparts. Basically this difference might be due to endocrine factors, the way they response to stressors, and even psychosocial burdens on females [34]. Furthermore, in this study, as students become older and older there was a relative increase in prevalence of headache even if it is not yet statistically associated which may be due to narrow age gaps within the samples. This agrees with other reports [3, 35, 36] which stated that the prevalence of headache rises from school age upwards.

Like sex, in this study students with family history of headache and lack of adequate vacation time were having higher prevalence of headache compared to their counterparts. Accordingly, the odds of having life time headache among students with family history of headache were 4.05 times more likely higher (AOR = 4.049; 95 % CI 2.465–6.653) than students without family history of headache. Supporting this reflection studies shown that headache do have genetic association [37–39]. Similarly, the likelihood of life time headache among students who didn’t have adequate vacation time was 3.86 times higher than their counterparts (AOR = 3.863; 95 % CI 2.235–6.677). This may be due to the fact that lack of free time and high workload may expose students for stressful life which may lead to induction or exacerbation of headache. Even if it is not statistically associated the prevalence of headache among year I (freshman) and medicine students is slightly higher than the total prevalence. For freshman students this might be due to difficulty of adapting the new educational and social environment that may lead to stressful situation. In case of medical student it might be due to high tension and stress subjected to them and even lack of adequate free time between courses/examinations.

The prevalence of migraine in this study was 13.06 % which is slightly higher than the previous community based studies in Ethiopia, 3–10 % [27, 40] and it is reported that migraine is prevalent in the urban than rural population [40]. But migraine prevalence in this study is closer to the global migraine prevalence 11 % [41], report from Benin 14.2 % [42], Turkey 12.4 % [23] and meta-analysis of community based studies of Africa 14.89 % [43]. Still for discrepancies methodological and study population difference, and the existing risk factors variation might be responsible. This study demonstrated that migraine prevalence among females (16.00 %) was higher than males (11.92 %) and found consistent with previous reports [43–45]. Supporting the genetic predisposition of migraine [38, 39] in this study 71 (75.53 %) of the migraineurs were having family history of headache.

In this student population stress, sleep disturbance and reading for a longer period were the top three triggering factors of headache and except due to study population differences this agrees with other reports [40, 46, 47]. The proper monitoring and understanding of triggering factors is the important footstep to lessen frequency and severity of headache and also its complications even if there is no adequate experimental support to associate each of them [48].

Among students who were experiencing headache in the last 12 months majority 360 (77.59 %) used self medications by either directly purchasing from pharmacies 208 (44.83 %) or taking drugs available from their own cabinet and/or share from their friends 152 (32.76 %). Such self medications may lead to frequent and high dose intake of analgesics and then this may also end up with analgesic over use headache and other untoward effects [49]. Only 32 (6.90 %) looked for medical care from physicians and the remaining 24 (5.17 %) used traditional treatment options like caffeinated drinks, chat and other substances. This low tendency of seeking medical care from physicians is comparable with reports of Heinisch [50] and Sanvito et al. [51]. But 120 (25.86 %) didn’t use drug to manage headache episodes rather they preferred to sleep or shower and take rest in a quiet place. Such nondrug treatment options of headache were also reported from other study [52] However, long lasting and frequent headache attacks without appropriate medical services might be a risk for other comorbidities [15] and low academic performance of students [53–55]. As it described in the Result section more than three fourth of students need to limit or avoid their daily activity during headache episodes at least rarely and this may be magnified by lack of appropriate therapy.

The life time drug use for the management of headache among students of this study was 429 (72.45 %) which has discrepancy compared to the school children analgesic use in southern Brazil [31]. The sociocultural and environmental factors, the level of education might be responsible for the discrepancy.

In this particular study, age, family history of headache, lack of adequate vacation time and migraine type of headache were found substantial factors of drug use for headache. Accordingly, females were 6.86 times (AOR = 6.859; 95 % CI 3.678–12.791) more likely to use drugs for treatment of headache as compared to male students. And this was consistent with many other studies [31, 56]. Similarly, the odds of using analgesics for headache among students whose age was 22–25 years were 2.43 times higher than students whose age was greater than 25 years. This might be due to high level of awareness of students on the adverse effects of drugs as they are being senior.

Students with a family history of headache 2.51 times more likely used drugs in their life time compared to those who didn’t have family history of headache. As it is described above most migraineur students were having family history of headache and thus, due to high severity of migraine and sharing of drug use experience from their parents might be responsible for high analgesic drug use prevalence among students having family history of headache. Furthermore, type of headache was also found the other important factor to determine level of drug use for headache among students. Thus, the odds of drug use for headache among migraineurs was 3.49 times higher than the tension type headache sufferers (AOR = 3.494; 95 % CI 1.589–7.686). In its descriptive form, among migraineurs 86 (91.49 %) and among tension type headache sufferers 337 (70.06 %) were having analgesic use experience. Obviously, this may be due to intolerance of students for migraine episodes which are often sever and disabling than tension type headache without taking pharmaceuticals [57]. Thus, to prevent possible risks of developing other physical and psychiatric comorbidities and then to not negatively impact quality of life managing headache rationally especially, migraine is worthwhile [58, 59].

As it is described in the Result section majority of students who were used drugs, 64.10 % commonly used paracetamol to treat their headache and about one fourth of them used diclofenac. The use of paracetamol in majority of students in this study is similar with the report of Mehuys et al. [60]. While it contradicts with data reported by Barea et al. [31] and Ray et al. [61] in which most of the headache sufferers used aspirin than paracetamol in nearly 6:1 ratio. This discrepancy of analgesic use pattern probably reflects the existence of experience difference on the utilization of analgesics among the communities and health care providers of different nations. In this study only 16 (18.61 %) drug user migraineurs commonly used migraine specific drugs during migraine attacks. This may be due to the fact that most of the subjects in this study used self medication to manage their headache and that may limit their exposure and understanding of new and effective analgesics.

Subjects of this study when they found the initial treatment of headache is infective or less effective some of them preferred to take either additional dose and/or analgesic. However, frequent and over consumption of analgesics is a known cause of hepatotoxicity, dependency, withdrawal syndromes, medication over use headache and others [49, 62–64]. Supporting all this frequent drug intake limitations subjects of this study perceived that frequent consumption of analgesics is a reason for dependency, relapsing/rebound headache, adaptation and some other untoward effects.

In this study there was no experience of prophylactic drug use for headache among students. However, in addition to reducing/preventing the triggering factors prophylactic medications are often recommendable for patients who have frequent and refractory type of headache and when the frequency of acute medication use is approaching levels that place the patient at risk for medication overuse headache [59].

### Limitation of the study

Related to its design this study has considerable limitations that should be kept in mind while interpreting the results. Thus, being cross sectional study makes it less suitable to determine definitive cause and effect associations. Except the diagnostic step, all data is based on self reporting hence the study might be affected by reporting bias. Furthermore, the study is also prone for recall bias since most of the questions require recalling of past experiences.

## Conclusion

There was high prevalence of primary headache and majority of students didn’t seek medical care but largely consuming analgesics to treat their headache. Stress/tension, sleep disturbance and reading for a long period of time were the top perceived triggering factors of headache among the students. Considering all these, headache is becoming the most disabling and challenging health problem in the student segment of population. As a result, the policy makers, academic officials, health professionals and all the other concerned bodies should give appropriate attention to design preventive and therapeutic strategies of primary headache.

## Abbreviations

ICHD: International Classification of Headache Disorders
IHS: International Headache Society
NSAIDs: Nonsteriodal Antiflammatory Drugs
OR: odds ratio
